# Metabolism of primaquine in normal human volunteers: investigation of phase I and phase II metabolites from plasma and urine using ultra-high performance liquid chromatography-quadrupole time-of-flight mass spectrometry

**DOI:** 10.1186/s12936-018-2433-z

**Published:** 2018-08-13

**Authors:** Bharathi Avula, Babu L. Tekwani, Narayan D. Chaurasiya, Pius Fasinu, N. P. Dhammika Nanayakkara, H. M. T. Bhandara Herath, Yan-Hong Wang, Ji-Yeong Bae, Shabana I. Khan, Mahmoud A. Elsohly, James D. McChesney, Peter A. Zimmerman, Ikhlas A. Khan, Larry A. Walker

**Affiliations:** 1 National Center for Natural Products Research, School of Pharmacy, The University of Mississippi, University, MS 38677 USA; 2 Ironstone Separations, Inc., 147 CR 245, Etta, MS 38627 USA; 3 Center for Global Health & Diseases, Case Western Reserve University Cleveland, Ohio, 44106 USA

**Keywords:** Primaquine, Antimalarial, Malaria, *Plasmodium vivax*, 8-aminoquinoline, Drug metabolism, UHPLC-QToF-MS, Cytochrome P_450_

## Abstract

**Background:**

Primaquine (PQ), an 8-aminoquinoline, is the only drug approved by the United States Food and Drug Administration for radical cure and prevention of relapse in *Plasmodium vivax* infections. Knowledge of the metabolism of PQ is critical for understanding the therapeutic efficacy and hemolytic toxicity of this drug. Recent in vitro studies with primary human hepatocytes have been useful for developing the ultra high-performance liquid chromatography coupled with high-resolution mass spectrometric (UHPLC-QToF-MS) methods for simultaneous determination of PQ and its metabolites generated through phase I and phase II pathways for drug metabolism.

**Methods:**

These methods were further optimized and applied for phenotyping PQ metabolites from plasma and urine from healthy human volunteers treated with single 45 mg dose of PQ. Identity of the metabolites was predicted by MetaboLynx using LC–MS/MS fragmentation patterns. Selected metabolites were confirmed with appropriate standards.

**Results:**

Besides PQ and carboxy PQ (cPQ), the major plasma metabolite, thirty-four additional metabolites were identified in human plasma and urine. Based on these metabolites, PQ is viewed as metabolized in humans via three pathways. Pathway 1 involves direct glucuronide/glucose/carbamate/acetate conjugation of PQ. Pathway 2 involves hydroxylation (likely cytochrome P450-mediated) at different positions on the quinoline ring, with mono-, di-, or even tri-hydroxylations possible, and subsequent glucuronide conjugation of the hydroxylated metabolites. Pathway 3 involves the monoamine oxidase catalyzed oxidative deamination of PQ resulting in formation of PQ-aldehyde, PQ alcohol and cPQ, which are further metabolized through additional phase I hydroxylations and/or phase II glucuronide conjugations.

**Conclusion:**

This approach and these findings augment our understanding and provide comprehensive view of pathways for PQ metabolism in humans. These will advance the clinical studies of PQ metabolism in different populations for different therapeutic regimens and an understanding of the role these play in PQ efficacy and safety outcomes, and their possible relation to metabolizing enzyme polymorphisms.

**Electronic supplementary material:**

The online version of this article (10.1186/s12936-018-2433-z) contains supplementary material, which is available to authorized users.

## Background

Primaquine (PQ), an 8-aminoquinoline, is the only drug approved by the US FDA for treatment and radical cure of relapsing *Plasmodium vivax* malaria [1]. The World Health Organization (WHO) has recently recommended use of a single low dose of primaquine for mass drug administration programmes for control and elimination of *Plasmodium falciparum* malaria [2], primarily due to action of PQ against mature *P. falciparum* gametocytes and prevention of malaria transmission [3]. Clinical utility of PQ has also been indicated for treatment of other infectious diseases, namely leishmaniasis, trypanosomiasis and pneumocystis pneumonia [4]. However, the use of PQ has been restricted due to toxic effects, especially haemolytic toxicity in individuals with genetic deficiency of glucose-6-phosphate dehydrogenase (G6PD) [5, 6]. G6PD deficiency is the most common enzymopathy with more than > 400 million cases world-wide [7, 8] and it is more prevalent in the malaria endemic areas [9]. The anti-malarial activity and toxicity of PQ have been attributed to one, or more, of its metabolites [10–12]. PQ is rapidly metabolized to the carboxyprimaquine (cPQ) by oxidative deamination [13]. Phenolic metabolites of PQ have long been suggested as potential haemotoxic species [12]. The oxidation products have been identified as metabolites of PQ in the biological fluids of experimental animals treated with PQ. These putative hydroxylated metabolites have been observed to cause oxidative damage to normal and G6PD-deficient erythrocytes [14–17].

Recent experimental studies with primary human hepatocytes [13, 18–20] and the recombinant human enzymes (CYPs and amine oxidases) [12, 21] have been useful in phenotyping of key PQ metabolites and their relative quantification. The results have been utilized for prediction of pathways for metabolism of PQ [12, 18, 22]. Metabolism of PQ has been presumed to follow two distinct pathways. A major pathway is thought to be initiated by oxidative deamination of PQ to cPQ [18, 23]. Carboxy PQ is further metabolized through CYP mediated pathways and phase II metabolism, generating quinone-imine metabolites and glucuronide conjugates [18, 22]. This pathway has been suggested to determine characteristic pharmacokinetic and pharmacodynamic properties of PQ [13, 24–27]. Another pathway, mediated through CYPs (predominantly CYP2D6), generates multiple hydroxylated metabolites [21]. CYP2D6-mediated oxidation of PQ occurs at different positions on the quinoline ring. PQ-5,6-ortho quinone was identified as the major CYP2D6 metabolite, formed by degradation of the unstable 5-hydroxy-PQ [21]. Most of the previous studies on clinical pharmacokinetics and metabolism of PQ have been limited to quantification of parent drug PQ and cPQ (the major plasma metabolite) [13, 23–27]. This study extends the investigation to other phase I and phase II metabolites of PQ from plasma and urine of normal human volunteers treated with a single dose of PQ (45 mg).

The diverse analytical methods focused on PQ or its analogues have so far targeted pharmacokinetic studies and metabolite analyses [28–30] or isomer separation/characterization [31]. The methods employing ultra-high performance chromatography coupled with high-resolution mass spectrometry offer significant advantages over other analytical methods including high speed and sensitivity. This approach was recently employed for phenotyping PQ metabolites generated in vitro on incubation of stable isotope (^13^C) labelled PQ with primary human hepatocytes [18, 20] or recombinant human CYP2D6 [21]. This approach was useful in developing UHPLC-QToF-MS method for simultaneous analysis of PQ and its metabolites in biological samples. This method has been further optimized for the analysis of PQ metabolites in human plasma and urine.

## Methods

### Chemicals and materials

Acetonitrile and methanol of HPLC-grade were obtained from Fisher Scientific (Fair Lawn, NJ, USA). Water was purified in a Milli-Q system (Millipore, Bedford, MA, USA). Primaquine diphosphate and formic acid were purchased from Sigma (St Louis, MO, USA). Carboxyprimaquine (cPQ) was prepared using the procedure reported by McChesney and Sarangan [32]. Primaquine alcohol, 2-hydroxyprimaquine, 3-hydroxyprimaquine, 4-hydroxyprimaquine, 5-hydroxyprimaquine, primaquine-5,6-*ortho*-quinone, primaquine *N*-carbamoyl glucuronide, primaquine methyl carbamate, carboxy primaquine lactam, carboxy primaquine methyl ester and primaquine *N*-acetate were synthesized at National Center for Natural Products Research (NCNPR) and their identity and purity were confirmed by spectral data (IR, NMR and High-Resolution MS) and comparison of their physical data (m.p.) with published values [20, 21].

### Subjects, treatment, samples collection

The study was conducted with seven healthy adult human volunteers (age 26–51 years). The information on age, sex and ethnicity of individual human volunteers are provided in a previous report [13]. The individuals were orally administered three tablets of primaquine phosphate (equivalent to a total dose of 45 mg primaquine base) (Sanofi-Aventis US), 30 min after a normal breakfast. Blood samples were collected in 9 mL heparin vacutainer^®^ tubes at different time intervals, ranging from 30 min to 24 h, after administration of PQ. The tubes with blood samples were immediately processed for centrifugation under refrigerated conditions (4 °C) and separation of plasma and erythrocytes pellet. The urine samples from each individual were collected before treatment and up to 24 h after the treatment. The plasma and urine samples from individual volunteers were divided into aliquots, kept on dry ice and transferred for storage at − 80 °C till further analysis. The aliquots of plasma and urine samples were processed and analysed using UHPLC-QToF-MS for analysis and phenotyping the PQ metabolites as described below.

### Sample preparation

*Plasma sample preparation:* Aliquots of 500 μL plasma samples from PQ treated normal human volunteers, stored at − 80 °C, were thawed on ice. The plasma was treated with 1 mL chilled methanol, vortexed and stored overnight at − 80 °C. The samples were withdrawn from the freezer, vortexed and kept in an ultrasonic water bath for 10 min. Later they were centrifuged for 10 min at 14,000 rpm. The clear supernatants were transferred to fresh tubes, evaporated to dryness in a vacuum centrifuge and individually reconstituted with 200 μL chilled HPLC grade methanol. The samples were transferred to HPLC sample vials for analysis.

#### Urine sample preparation

Aliquots of 10 mL of urine samples were freeze-dried. The dried samples were reconstituted with 1 mL chilled HPLC grade methanol. The samples were kept on an ultrasonic water bath, vortexed and centrifuged at 14,000 rpm for 10 min. The clear supernatants were transferred to fresh vials and subjected to analysis.

### Metabolite identification using UHPLC-QToF-MS

The UHPLC-QToF-MS analytical method and other conditions were as described in earlier publications from us [20, 21]. Plasma and urine samples were analysed using a Waters Acquity UPLC™ system (Waters Corp., Milford, MA, USA) coupled to a Xevo Q-ToF mass spectrometer. Chromatographic separations were achieved using BEH C18 column (2.1 mm × 100 mm, 1.7 μm) at a flow rate of 0.25 mL/min. MS conditions were optimized in the positive electrospray mode with the instrumental parameters. Ten microlitre of sample was injected. PQ and metabolites were assigned with respect to the mass of the compounds and comparison of the retention times. This method involved the use of [M + H]^+^ ions of the compound PQ which was observed in the positive ion mode at *m/z* 260.1774 (calculated *m/z* = 260.1763) and for PQ metabolites (carboxyprimaquine, 2-hydroxyprimaquine, 3-hydroxyprimaquine, 4-hydroxy-primaquine, primaquine *N*-carbamoyl glucuronide, primaquine methyl carbamate, carboxy primaquine lactam, carboxy primaquine methyl ester and primaquine *N*-acetate). Prior to performing all experiments, the instrument was calibrated in positive ionization mode over a 50-1000 Da mass range, using a 2 µg/µL solution of sodium formate dissolved in methanol. This solution produces singly charged reference ions in both ion modes and is ideal as a calibrant for low molecular weight analysis. Further, the fragmentation patterns observed in the mass spectrum were useful in characterization of the compound. MetaboLynx™ processing software (Waters Corp., Manchester, UK) was used to assist molecular ion detection and a combination of accurate mass and MS/MS data on the molecular ions was used for structural identification of individual metabolites.

## Results

The PQ metabolites in plasma and urine were identified based on the retention time, HR-MS and MS/MS fragmentation patterns of individual metabolites. The formation and identity of metabolites were supported by their accurate mass and fragmentation pattern. The chemical structures of these metabolites were predicted by the MetaboLynx XS, a software algorithm for metabolite prediction, based on their MS–MS fragmentation ions as described below and other associated information (Table 1) (Additional file 1). The proposed fragmentation patterns and other associated information for PQ and other metabolites provided information about their structural skeleton. Besides PQ (*m/z* 260.1769) (**15**) and cPQ (*m/z* 275.1385) (**33**) (Table 1), thirty-four PQ metabolites, whose structures could be determined with MS/MS fragmentation ions, were detected in the plasma and urine samples. MS/MS fragmentation profiles for the selected metabolites, determined with confidence, are presented in the supplement data (Additional file 1). Molecular characteristics of individual metabolites are described below and also are presented in Table 1. The metabolites are listed in order of their retention time and detection in both plasma & urine, only in plasma and only in urine.

**Table 1:** HR-MS data for primaquine and its metabolites from human plasma and urine samples

#	RT (min)	Exact mass	Peak^a^ [M + H]^+^	Molecular Formula	Biotransformation (Metabolite Predicted/Identified)	Sample/source
**1**	3.5	273.1477	274.1549 (274.1550)	C_15_H_19_N_3_O_2_	Hydroxylation and quinone-imine formation	Plasma/urine
**2**	4.97	259.1679	260.1769 (260.1757)	C_15_H_21_N_3_O	Precursor ion (PQ)	
**3**	5.3	256.1206	257.1273 (257.1285)	C_15_H_16_N_2_O_2_	Oxidative deamination to acid + cyclization (Carboxyprimaquine lactam)	
**4**	7.98	479.1898	480.1992 (480.1977)	C_22_H_29_N_3_O_9_	Carbamoyl glucuronide formation (PQ-*N*-carbamoyl-glucuronide conjugate)	
**5**	8.95	274.1312	275.1385 (275.1390)	C_15_H_18_N_2_O_3_	Deamination + Acid (Carboxyprimaquine)	
**6**	10.12	288.1468	289.1560 (289.1547)	C_16_H_20_N_2_O_3_	Deamination + Acid + Methylation (Carboxyprimaquine methyl ester)	
**7**	4.67	421.2207	422.2315 (422.2286)	C_21_H_31_N_3_O_6_	Glucose conjugation	Plasma
**8**	6.03	320.1367	321.1454 (321.1445)	C_16_H_20_N_2_O_5_	(Deamindation + acid) + 2xOH + methylation (dihydroxy carboxyprimaquine methyl ester)	
**9**	9.65	317.1734	318.1789 (318.1812)	C_17_H_23_N_3_O_3_	Methyl carbamate formation (PQ methyl carbamate)	
**10**	1.78	259.1315	260.1403 (260.1399)*	C_14_H_17_N_3_O_2_	5-Hydroxylation +Orthoquinone formation (5,6 orthoquinone primaquine)	Urine
**11**	1.9	451.1955	452.2011 (452.2027)	C_21_H_29_N_3_O_8_	Hydroxylation + Glucuronide conjugation	
**12**	2.2	421.1849	422.1908 (422.1922)	C_20_H_27_N_3_O_7_	5-Demethylation + Glucuronidation (5-desmethyl PQ glucuronide)	
**13**	2.32	451.1955	452.2011 (452.2027)	C_21_H_29_N_3_O_8_	Hydroxylation + Glucuronide conjugation	
**14**	2.34	275.1634	276.1712 (276.1707)	C_15_H_21_N_3_O_2_	Hydroxylation (4-OH PQ)	
**15**	3.1	452.1795	453.1881 (453.1868)	C_21_H_28_N_2_O_9_	Deamination + alcohol + hydroxylation + glucuronidation	
**16**	3.12	315.1947	316.2002 (316.2020)	C_18_H_25_N_3_O_2_	Methylation + Acetylation	
**17**	3.45	275.1634	276.1694 (276.1707)	C_15_H_21_N_3_O_2_	Hydroxylation	
**18**	3.85	275.1634	276.1697 (276.1707)	C_15_H_21_N_3_O_2_	Hydroxylation (2-OH PQ)	
**19**	4.43	245.1528	246.1617 (246.1601)	C_14_H_19_N_3_O	Demethylation	
**20**	4.88	493.206	494.2127 (494.2133)	C_23_H_31_N_3_O_9_	Acetylation + (Hydroxylation + Glucuronide conjugation)	
**21**	4.92	275.1634	276.1705 (276.1707)	C_15_H_21_N_3_O_2_	Hydroxylation (3-OH PQ)	
**22**	5.18	273.1477	274.1534 (274.1550)	C_15_H_19_N_3_O_2_	Hydroxylation and quinone-imine formation	
**23**	5.67	483.1853	484.1932 (484.1926)	C_21_H_29_N_3_O_10_	3 x Hydroxylation + 1 x glucuronide conjugation	
**24**	5.9	289.1426	290.1500 (290.1499)	C_15_H_19_N_3_O_3_	2 x Hydroxylation + quinone-imine formation	
**25**	5.92	291.1583	292.1647 (292.1656)	C_15_H_21_N_3_O_3_	2 x hydroxylation	
**26**	6.15	498.1486	499.1545 (499.1559)	C_21_H_26_N_2_O_12_	Deamination + acid + 3 x hydroxylation + 1 x glucuronide conjugate	
**27**	6.17	466.1587	467.1666 (467.1660)	C_21_H_26_N_2_O_10_	(Hydroxylation + glucuronide conjugation) + (deamination + acid)	
**28**	6.4	291.1583	292.1647 (292.1656)	C_15_H_21_N_3_O_3_	2 x hydroxylation	
**29**	7.27	301.179	302.1875 (302.1863)	C_17_H_23_N_3_O_2_	Acetylation	
**30**	7.6	435.2006	436.2100 (436.2078)	C_21_H_29_N_3_O_7_	Glucuronide conjugation	
**31**	7.74	436.1846	437.1909 (437.1918)	C_21_H_28_N_2_O_8_	Oxidative deamination to alcohol + glucuronide conjugation	
**32**	7.79	450.1638	451.1714 (451.1711)	C_21_H_26_N_2_O_9_	(Deamination + acid) + glucuronide conjugation	
**33**	8.2	276.1474	277.1552 (277.1547)	C_15_H_20_N_2_O_3_	Deamination + alcohol + hydroxylation	
**34**	8.6	435.2006	436.2100 (436.2078)	C_21_H_29_N_3_O_7_	Glucuronide conjugation	
**35**	8.65	315.1583	316.1676 (316.1656)	C_17_H_21_N_3_O_3_	Acetylation + hydroxylation + quinone-imine formation	
**36**	10.38	302.1267	303.1324 (303.1339)	C_16_H_18_N_2_O_4_	Deamination + acid + methylation + hydroxylation + quinone-imine formation	

^a^Observed mass of the metabolite (calculated mass is shown in parenthesis)

### Primaquine quinone-imine (**1**,**22**)

ESI-HRMS of this metabolite gave a molecular ion peak at *m/z* 274.1549 [M + H]^+^, corresponds to the molecular formula C_15_H_20_N_3_O_2_. Key fragments detected by 274 peak in the LC-QToF-MS spectra are 260.1365 [M + H-CH_2_], 243.1127 [M + H-CH_3_O]^+^, 241.1363 [M + H-H_3_NO]^+^, 145.0547 [M + H-C_6_H_13_N_2_O]^+^ and 86.0964 [M + H-C_10_H_8_N_2_O_2_]^+^ (Additional file 1) (Table 1). Based on retention time, accurate mass and fragmention ions, these metabolites were predicted as a PQ quinone-imine, likely to be generated by hydroxylation of PQ and then dehydrogenation (Fig. 2). Identification of two PQ quinone-imine metabolites with identical MS/MS profile and different retention times likely indicates hydroxylation at different positions on the quinolone ring.

### Primaquine (**2**)

ESI-HRMS of PQ in plasma and urine gave a molecular ion peak at *m/z* 260.1769 [M + H]^+^ which corresponded to the molecular formula C_15_H_22_N_3_O. The MS/MS spectrum of precursor ion, PQ showed fragments at *m*/*z* 243.1521 (−17; –NH_3_, loss of ammonia) confirming the presence of amino group in the molecule, 175.0867 (−85; –C_5_H_11_N), 86.0974 (−174; –C_10_H_10_N_2_O) (Additional file 1) (Table 1) and was identical to the MS/MS of synthetic PQ with the same retention time.

### Carboxy-primaquine lactam (**3**)

ESI-HRMS of this metabolite gave a molecular ion peak at *m/z* 257.1273 [M + H]^+^ which corresponds to the molecular formula C_15_H_17_N_2_O_2_. Key fragments detected by 257.1273 peak in the LC-QTOF-MS spectra are 239.1151 [M + H-H_2_O]^+^, 175.0867 [M + H-C_5_H_6_O]^+^, 83.0474 [M + H-C_10_H_10_N_2_O]^+^ (Additional file 1) (Table 1). The metabolite is likely to be formed by oxidative de-amination of PQ followed by cyclization of the aldehyde form and was identical to the MS/MS of synthetic carboxy-primaquine lactam with the same retention time (Fig. 3).

### Primaquine *N*-carbamoyl glucuronide (**4**)

**Fig. 1:**
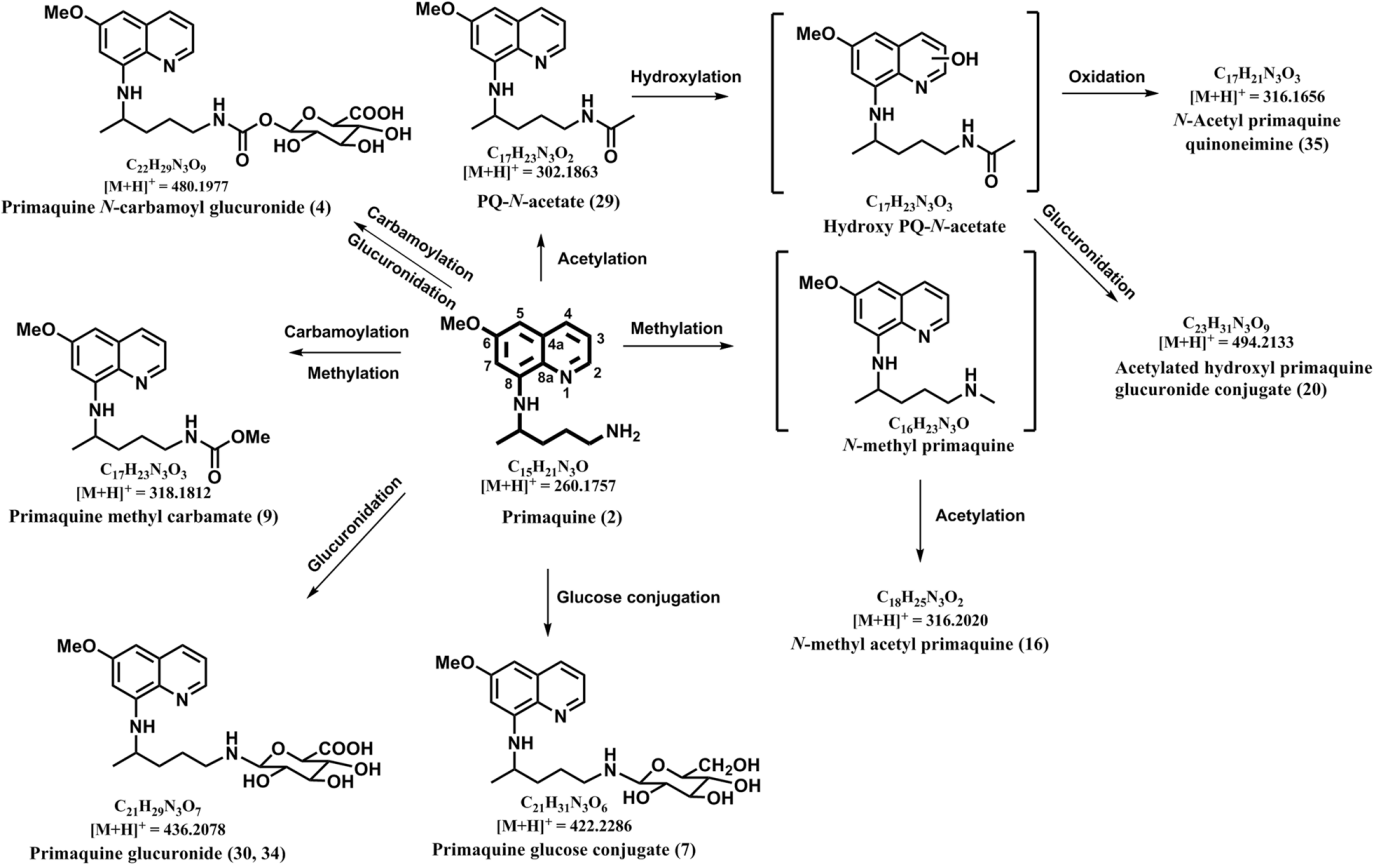
Pathway for human metabolism of PQ—pathway 1: direct metabolism of PQ through phase II conjugation reactions

ESI-HRMS of this metabolite gave a molecular ion peak at *m/z* 480.1992 [M + H]^+^ which corresponds to the molecular formula C_22_H_29_N_3_O_9_. Key fragments detected by 480 peak in the LC-QToF-MS spectra are *m/z* 436.2118 [M + H-CO_2_]^+^, 304.1658 [M + H-C_6_H_8_O_6_]^+^, 260.1767 [M + H-C_7_H_8_O_8_]^+^, 243.1500 [M + H-C_7_H_11_NO_8_]^+^, 175.0877 [M + H-C_12_H_19_NO_8_]^+^, 86.0972 [M + H-C_7_H_8_O_8_C_10_H_10_N_2_O]^+^ (Additional file 1) (Table 1). This metabolite is likely to be formed due to the carbamoylation of PQ followed by glucuronide formation and was identical to the MS/MS of synthetic primaquine *N*-carbamoyl glucuronide with the same retention time (Fig. 1).

### Carboxyprimaquine (**5**)

ESI-HRMS of this metabolite gave a molecular ion peak at *m/z* 275.1385 [M + H]^+^ which corresponded to the molecular formula C_15_H_19_N_2_O_3_. The side chain of PQ undergoes biotransformation to PQ-aldehyde (oxidative deamination) and then converted to an acid (carboxyprimaquine, cPQ) by an aldehyde dehydrogenase. Key fragments detected by cPQ are 257.1306 [M + H-H_2_O]^+^ (intense peak), 175.0842 [M + H-C_5_H_8_O_2_]^+^, 101.0593 [M + H-C_10_H_10_N_2_O]^+^, 83.0493 [M + H-C_10_H_12_N_2_O_2_]^+^ (Additional file 1) (Table 1). The MS/MS spectrum of precursor ion carboxy primaquine showed five fragment ions and was identical to the MS/MS of synthetic cPQ with the same retention time. This metabolic pathway for cPQ is presented in Fig. 3.

### Carboxy primaquine methyl ester (**6**)

ESI-HRMS of this metabolite gave a molecular ion peak at *m/z* 289.1560 [M + H]^+^ which corresponds to the molecular formula C_16_H_21_N_2_O_3_. The biotransformation is due to methyl carbamate formation. Key fragments detected by *m/z* 289 peak in the LC-QToF-MS spectra are *m/z* 257.1287 [M + H-CH_3_OH]^+^, 175.0843 [M + H-C_6_H_10_O_2_]^+^, 83.048 [M + H-C_11_H_14_N_2_O_2_]^+^ (Additional file 1) (Table 1). Formation of this metabolite is likely to occur through formation of PQ-aldehyde (oxidative deamination) followed by conversion to an acid (carboxy primaquine) and subsequent methylation (Fig. 3). The LC–MS/MS profile of this metabolite was identical to the MS/MS of synthetic carboxyprimaquine methyl ester with the same retention time.

### Primaquine-glucose conjugate (**7**)

ESI-HRMS of this metabolite gave a molecular ion peak at *m/z* 422.2315 [M + H]^+^ which corresponded to the molecular formula C_21_H_32_N_3_O_6_. The MS/MS spectrum of precursor ion PQ-glucose showed fragments at *m*/*z* 404.2209 (−18; –H_2_O), 260.177 (−162; –C_6_H_10_O_5_), 243.1523 (−179; –C_6_H_13_NO_5_), 175.0895 (−247; –C_11_H_21_NO_5_), 86.0959 (−336; –C_16_H_20_N_2_O_6_) (Additional file 1) (Table 1), and was identical to the MS/MS of synthetic PQ-glucose with the same retention time. The biotransformation is due to PQ conjugation with a glucose moiety (Fig. 1).

### Dihydroxy-carboxy primaquine methyl ester (**8**)

ESI-HRMS of this metabolite gave a molecular ion peak at *m/z* 321.1454 [M + H]^+^ which corresponds to the molecular formula C_16_H_21_N_2_O_5_ (Table 1). This metabolite was detected in very low quantities and the identity could not be established with confidence. Tentative identity was predicted as dihydoxy-carboxy primaquine methyl ester, generated through double hydroxylations on the quinolone ring followed methylation at side chain carboxy group (Fig. 3).

### Primaquine methylcarbamate (**9**)

ESI-HRMS of this metabolite gave a molecular ion peak at *m/z* 318.1789 [M + H]^+^ which corresponds to the molecular formula C_17_H_24_N_3_O_3_. Key fragments detected by *m/z* 318 peak in the LC-QToF-MS spectra are *m/z* 175.0853 [M + H-C_7_H_13_NO_2_]^+^ (intense peak), 160.0619 [M + H-C_8_H_16_NO_2_]^+^, 88.0384 [M + H-C_14_H_18_N_2_O]^+^ (Additional file 1) (Table 1). This metabolite is likely to be generated by carbamoylation of PQ followed by methyl carbamate formation and was identical to the MS/MS of synthetic primaquine methylcarbamate with the same retention time (Fig. 1).

### Primaquine-5,6-*ortho*-quinone (**10**)

**Fig. 2:**
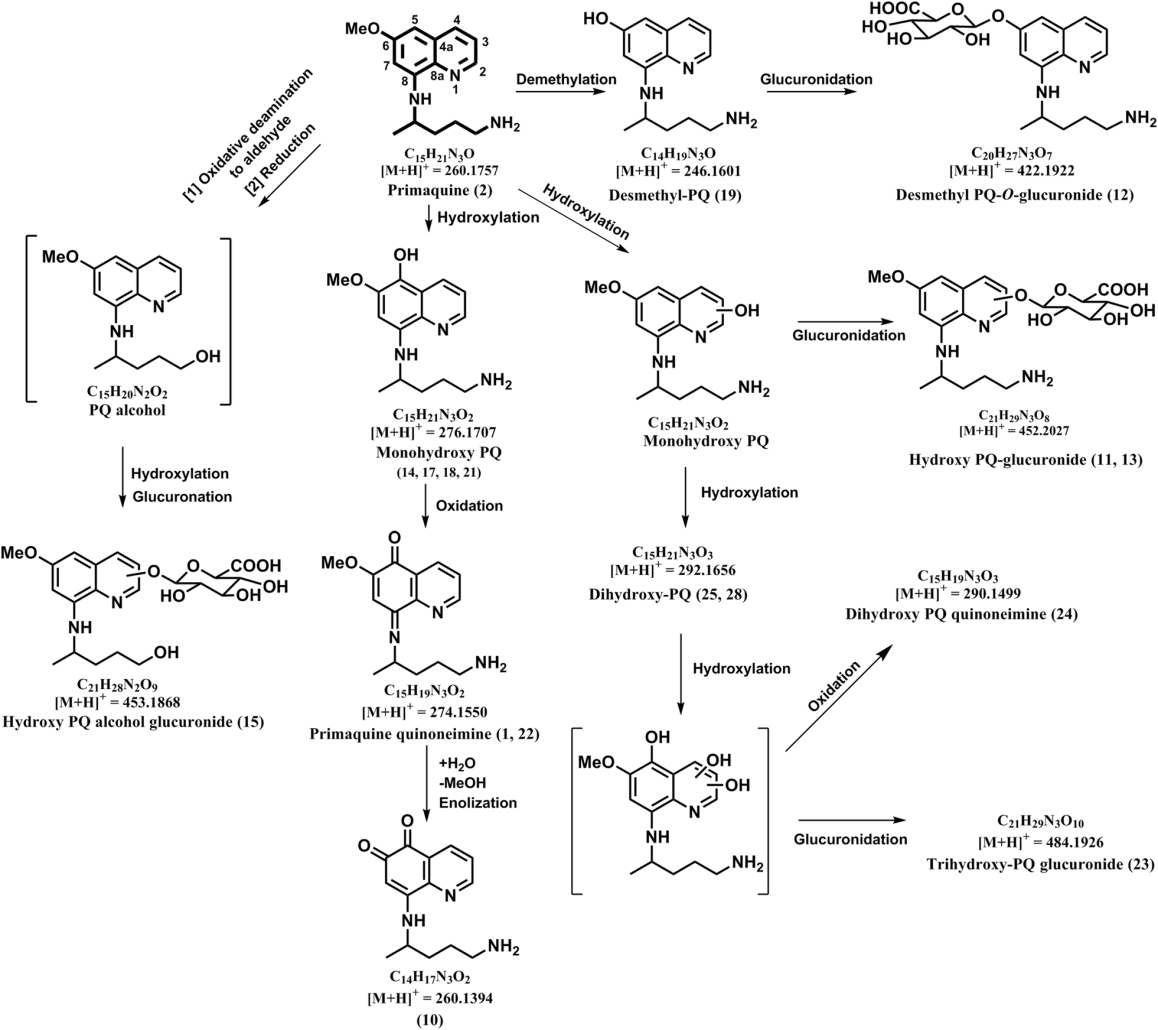
Pathway for human metabolism of PQ—pathway 2: metabolism of PQ through phase I ring hydroxylations followed by phase II conjugations

ESI-HRMS of this metabolite gave a molecular ion peak at *m/z* 260.1403 [M + H]^+^ which corresponds to the molecular formula C_14_H_18_N_3_O_2_. Key fragments detected by 260 peak in the LC-QToF-MS spectra of all samples are 243.1134 [M + H-NH_3_]^+^, 215.1166 [M + H-CH_3_NO]^+^, 175.0508 [M + H-C_5_H_11_N]^+^ (intense peak), 147.0553 [M + H-C_6_H_11_NO]^+^, 86.0970 [M + H-C_9_H_6_N_2_O_2_]^+^ (Additional file 1) (Table 1). The metabolite is generated by hydroxylation of PQ, followed by the quinone-imine formation, with further oxidation and effective loss of the methyl group at the 6 position (Fig. 2). It was identical to the MS/MS of synthetic primaquine-5,6-*ortho*-quinone with the same retention time.

### Hydroxy-primaquine glucuronide (**11**, **13**)

ESI-HRMS of this metabolite gave a molecular ion peak at *m/z* 452.2011 [M + H]^+^ (Table 1) which corresponds to the molecular formula C_21_H_30_N_3_O_8_. Based on retention time, accurate mass and fragment ions, this metabolite was predicted as hydroxy-primaquine glucuronide likely to be generated by hydroxylation, followed by glucuronide conjugation of PQ (Fig. 2). Identification of two metabolites with identical MS/MS profile but different retention times indicates hydroxylation at different positions on the quinoline ring.

### Desmethyl-primaquine-*O*-glucuronide (**12**)

ESI-HRMS of this metabolite gave a molecular ion peak at *m/z* 422.1908 [M + H]^+^ which corresponds to the molecular formula C_20_H_28_N_3_O_7_. Key fragments detected by *m/z* 422 peak in the LC-QToF-MS spectra of all samples are 405.1536 [M + H-CH_5_]^+^, 246.1606 [M + H-C_6_H_8_O_6_]^+^, 229.1341 [M + H-C_6_H_10_NO_6_]^+^, 86.0970 [M + H-C_15_H_16_N_2_O_7_]^+^, 69.0704 [M + H-C_15_H_19_N_3_O_7_]^+^ (Additional file 1) (Table 1). This metabolite was tentatively identified as desmethyl-primaquine-*O*-glucuronide based on accurate mass and fragments ions and was likely to be generated due to demethylation and glucuronide conjugation of PQ (Fig. 2).

### Monohydroxy-primaquine (**14**, **17**, **18**, **21**)

ESI-HRMS of these four metabolites gave identical molecular ion peak at *m/z* 276.1712 [M + H]^+^ which corresponds to the molecular formula C_15_H_22_N_3_O_2_. Key fragments detected by 276 peak in the LC-QToF-MS spectra of all samples are 259.1447 [M + H-NH_3_]^+^, 191.0821 [M + H-C_5_H_11_N]^+^, 86.0970 [M + H-C_10_H_10_N_2_O_2_]^+^ (Additional file 1) (Table 1). The three monohydroxy PQ metabolites are generated due to hydroxylation of PQ and were identical to the MS/MS of synthetic hydroxy-PQs namely, 4-hydroxy PQ (**14**), 2-Hydroxy PQ (**18**) and 4-hydroxy PQ (**19**) with the same retention time (Table 1) (Fig. 2). The exact identity, regarding position of hydroxylation on the quinoline ring, of fourth monohydroxylated PQ metabolite (**17**) could not be established due to non-availability of corresponding synthetic standard.

### Hydroxy-primaquine-alcohol-glucuronide (15)

**Fig. 3:**
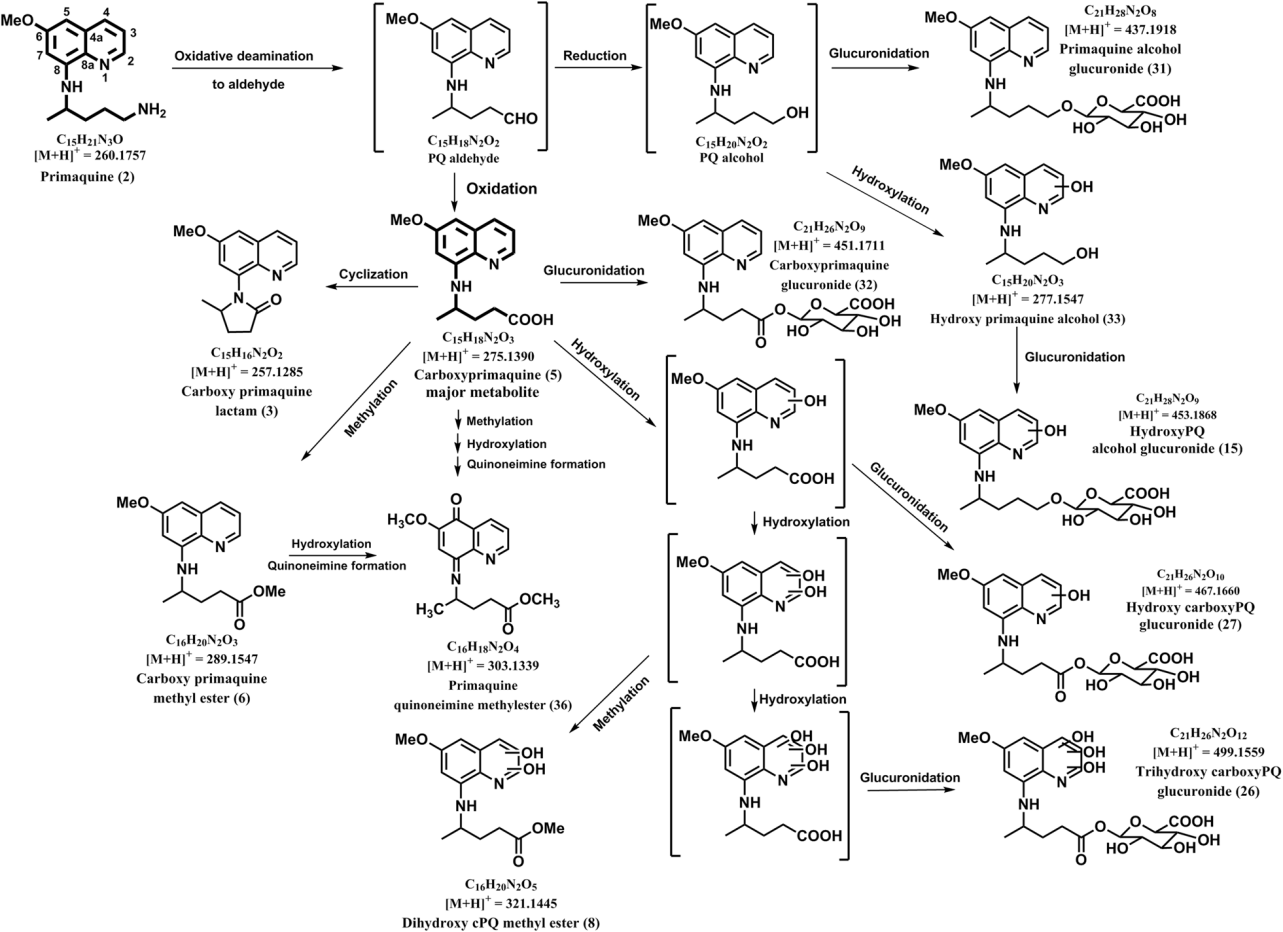
Pathway for human metabolism of PQ—pathway 3: metabolism of PQ through oxidative deamination 8-*N* alkylamino side chain followed by phase I ring hydroxylations and/or phase II conjugations

ESI-HRMS of this metabolite gave a molecular ion peak at *m/z* 453.1881 [M + H]^+^ which corresponds to the molecular formula C_21_H_29_N_2_O_9_. (Table 1). This metabolite was detected in very low quantities and the identity could not be established with confidence. Tentative identity was predicted as hydroxy-primaquine-alcohol-glucuronide. This metabolite is predictably generated through hydroxylation of PQ alcohol followed by glucuronide conjugation (Fig. 3).

### Acetylated *N*-methyl primaquine (**16**)

ESI-HRMS of this metabolite gave a molecular ion peak at *m/z* 316.2002 [M + H]^+^ which corresponds to the molecular formula C_18_H_26_N_3_O_2_ (Table 1). This metabolite was detected in very low quantities and the identity could not be established with confidence. Tentative identity was predicted as acetylated *N*-methyl primaquine. This metabolite is predictably generated through methylation of PQ and followed by acetylation (Fig. 1).

### 6-desmethyl-primaquine (**19**)

ESI-HRMS of this metabolite gave a molecular ion peak at *m/z* 246.1617 [M + H]^+^ which corresponded to the molecular formula C_14_H_20_N_3_O. Key fragments detected by *m/z* 246 peak in the LC-QToF-MS spectra are 229.1293 [M + H-NH_3_]^+^ (intense peak), 161.0751 [M + H-C_5_H_11_N]^+^, 86.0966 [M + H-C_9_H_8_N_2_O]^+^, 69.0721 [M + H-C_9_H_11_N_3_O]^+^ (Additional file 1) (Table 1). The metabolite was tentatively identified as 6-desmethyl PQ based on accurate mass and fragments ions. Demethylation of 6-methoxy group in PQ is likely to generate this 6-hydroxy metabolite of PQ (Fig. 2).

### Acetylated hydroxy-primaquine glucuronide conjugate (**20**)

ESI-HRMS of this metabolite gave a molecular ion peak at *m/z* 494.2127 [M + H]^+^ which corresponded to the molecular formula C_23_H_32_N_3_O_9_. Key fragments detected by *m/z* 494 peak in the LC-QToF-MS spectra are 318.1797 [M + H-C_6_H_8_O_6_]^+^, 191.0805 [M + H-C_13_H_21_NO_7_]^+^, 128.1067 [M + H-C_16_H_18_N_2_O_8_]^+^ and 86.0989 [M + H-C_18_H_20_N_2_O_9_]^+^ (Additional file 1) (Table 1). The metabolite was tentatively identified as acetylated hydroxy-primaquine glucuronide conjugate based on accurate mass and fragments ions and may be generated through PQ acetylation followed by monohydroxylation and conjugation with glucuronide moiety (Fig. 1).

### Trihydroxy-primaquine glucuronide (**23**)

ESI-HRMS of this metabolite gave a molecular ion peak at *m/z* 484.1932 [M + H]^+^ (Table 1) which corresponds to the molecular formula C_21_H_30_N_3_O_10_. This metabolite was detected in very low quantities and the identity could not be established with confidence. Tentative identity was predicted as trihydroxy-primaquine glucuronide (Fig. 2). The positions for tri hydroxylations on the quinoline ring could not be established due to non-availability of corresponding synthetic standards and very low quantity of the metabolite.

### Dihydroxy-primaquine quinone-imine (**24**)

ESI-HRMS of this metabolite gave a molecular ion peak at *m/z* 290.1500 [M + H]^+^ (Additional file 1) (Table 1) which corresponds to the molecular formula C_15_H_20_N_3_O_3_. Key fragments detected by *m/z* 290 peak in the LC-QToF-MS spectra are 273.1194 [M + H-NH_3_]^+^, 205.0590 [M + H-C_5_H_11_N]^+^, 191.0481 [M + H-C_6_H_13_N]^+^, 86.0955 [M + H-C_10_H_8_N_2_O_3_]^+^. The metabolite was tentatively identified as dihydroxy-primaquine quinone-imine based on accurate mass and fragments ions. This metabolite may be generated through two hydroxylations of primaquine, followed by oxidation to quinone imine (Fig. 2). The positions of hydroxylations in this metabolite could not be established due to non-availability of corresponding synthetic standards.

### Dihydroxy-primaquine (**25**, **28**)

ESI-HRMS of these metabolites gave a molecular ion peak at *m/z* 292.1647 [M + H]^+^ (Additional file 1) (Table 1) which corresponds to the molecular formula C_15_H_22_N_3_O_3_. Key fragments detected by *m/z* 292 peak in the LC-QToF-MS spectra are 275.1396 [M + H-NH_3_]^+^, 207.0770 [M + H-C_5_H_11_N]^+^, 87.1040 [M + H-C_10_H_9_N_2_O_3_]^+^. The two metabolites were tentatively identified as dihydroxy-primaquine based on accurate mass and fragments ions. These metabolites may be generated through two hydroxylations of primaquine (Fig. 2). The positions of hydroxylations in the individual metabolites could not be established due to non-availability of corresponding synthetic standards.

### Trihydroxy-carboxyprimaquine glucuronide (**26**)

ESI-HRMS of this metabolite gave a molecular ion peak at *m/z* 499.1545 [M + H]^+^ which corresponded to the molecular formula C_21_H_27_N_2_O_12_. Key fragments detected by 499 peak in the LC-QToF-MS spectra are 290.0903 [M + H-C_7_H_13_O_7_]^+^, 273.0875 [M + H-C_7_H_14_O_8_]^+^, 217.1076 [M + H-C_11_H_10_N_2_O_7_]^+^, 85.0653 [M + H-C_16_H_18_N_2_O_11_]^+^ (Additional file 1) (Table 1). This metabolite was tentatively identified as trihydroxy-carboxyprimaquine glucuronide based on accurate mass and fragments ions. This metabolite may be generated through three hydroxylations of carboxy primaquine followed by glucuronidation (Fig. 3). The positions of hydroxylations and site of glucuronidation in the individual metabolites could not be established due to non-availability of corresponding synthetic standards.

### Hydroxy-carboxyprimaquine glucuronide (**27**)

ESI-HRMS of this metabolite gave a molecular ion peak at *m/z* 467.1666 [M + H]^+^ which corresponded to the molecular formula C_21_H_27_N_2_O_10_. Key fragments detected by 467 peak in the LC-QToF-MS spectra are 449.1560 [M + H-H_2_O]^+^, 290.0876 [M + H-C_10_H_11_NO_2_]^+^, 273.1234 [M + H-C_6_H_10_O_7_]^+^, 217.1076 [M + H-C_11_H_10_N_2_O_5_]^+^, 191.0821 [M + H-C_11_H_16_O_8_]^+^ (Additional file 1) (Table 1). This metabolite was tentatively identified as hydroxy-carboxyprimaquine glucuronide based on accurate mass and fragments ions. This metabolite may be generated through hydroxylation of carboxy primaquine followed by glucuronidation (Fig. 3). The position of hydroxylation and site of glucuronidation in the individual metabolite could not be established due to non-availability of corresponding synthetic standard.

### Primaquine acetate (**29**)

ESI-HRMS of this metabolite gave a molecular ion peak at *m/z* 302.1875 [M + H]^+^ which corresponded to the molecular formula C_17_H_24_N_3_O_2_. Key fragments detected by 302 peak in the LC-QToF-MS spectra are *m/z* 243.1502 [M + H-C_2_H_5_NO]^+^, 175.0864 [M + H-C_7_H_13_NO]^+^, 128.1077 [M + H-C_10_H_10_N_2_O]^+^ (intense peak), 86.0966 [M + H-C_12_H_12_N_2_O_2_]^+^ (Additional file 1) (Table 1). This metabolite may be generated through acetylation of PQ to PQ *N*-acetate (Fig. 1) and was identical to the MS/MS of synthetic PQ acetate with the same retention time.

### Primaquine glucuronide (**30**, **34**)

ESI-HRMS of these metabolites gave a molecular ion peak at *m/z* 436.2100 [M + H]^+^ (Table 1), which corresponded to the molecular formula C_21_H_30_N_3_O_7_. These metabolites were detected in very low quantities and their identities could not be established with confidence. Tentative identity of these metabolites was predicted as PQ glucuronide (Fig. 1).

### Primaquine alcohol glucuronide (**31**)

ESI-HRMS of this metabolite gave a molecular ion peak at *m/z* 437.1909 [M + H]^+^ which corresponded to the molecular formula C_21_H_29_N_2_O_8_. Key fragments detected by PQ alcohol glucuronide in the LC-QToF-MS spectra of all samples are *m/z* 261.1598 [M + H-C_6_H_8_O_6_]^+^, 175.0868 [M + H-C_11_H_18_O_7_]^+^, 159.0310 [M + H-C_15_H_22_N_2_O_3_]^+^, 87.0810 [M + H-C_16_H_18_N_2_O_7_]^+^ (Additional file 1) (Table 1). This metabolite was tentatively identified as primaquine alcohol glucuronide based on accurate mass and fragments ions. This metabolite may be generated through oxidative de-amination of PQ to alcohol followed conjugation with glucuronide moiety (Fig. 3).

### Carboxyprimaquine glucuronide (**32**)

ESI-HRMS of this metabolite gave a molecular ion peak at *m/z* 451.1714 [M + H]^+^ which corresponds to the molecular formula C_21_H_27_N_2_O_9_. Key fragments detected by 451 peak in the LC-QToF-MS spectra are 275.1408 [M + H-C_6_H_8_O_6_]^+^, 257.1290 [M + H-C_6_H_10_O_7_]^+^ (intense peak), 175.0884 [M + H-C_11_H_16_O_8_]^+^, 101.0593 [M + H-C_16_H_18_N_2_O_7_]^+^ (Additional file 1) (Table 1). This metabolite may be generated through glucuronide conjugation of carboxy PQ and was identical to the MS/MS of synthetic carboxyprimaquine glucuronide with the same retention time (Fig. 3).

### Hydroxy-primaquine alcohol (**33**)

ESI-HRMS of this metabolite gave a molecular ion peak at *m/z* 277.1552 [M + H]^+^ which corresponds to the molecular formula C_15_H_21_N_2_O_3_. Key fragments detected by 277 peak in the LC-QToF-MS spectra are *m/z* 175.0871 [M + H-C_5_H_10_O_2_]^+^, 83.0797 [M + H-C_10_H_12_NO_3_]^+^ (Additional file 1) (Table 1). This metabolite was tentatively identified as hydroxyl primaquine alcohol based on accurate mass and fragments ions. This metabolite may be generated through oxidative de-amination of PQ to alcohol followed by hydroxylation (Fig. 3). However, the site for hydroxylation on the quinoline ring could not be established due to non-availability of corresponding synthetic standards.

### *N*-Acetyl-primaquine quinone-imine (**35**)

ESI-HRMS of this metabolite gave a molecular ion peak at *m/z* 316.1676 [M + H]^+^ which corresponds to the molecular formula C_17_H_22_N_3_O_3_. Key fragments detected by 316 peak in the LC-QToF-MS spectra are *m/z* 302.1402 [M + H-CH_2_]^+^, 147.0922 [M + H-C_8_H_11_NO_3_]^+^ (Additional file 1), (Table 1). This metabolite was tentatively identified as *N*-acetyl-primaquine quinone-imine based on accurate mass and fragments ions and may be generated through *N*-acetylation on the side chain and hydroxylation of PQ followed by oxidation to quinone-imine (Fig. 1).

### Primaquine quinone-imine carbamate (**36**)

ESI-HRMS of this metabolite gave a molecular ion peak at *m/z* 303.1321 [M + H]^+^ which corresponds to the molecular formula C_16_H_19_N_2_O_4_. Key fragments detected by 303 peak in the LC-QToF-MS spectra are *m/z* 287.1365 [M + H–O]^+^, 271.1016 [M + H-CH_3_OH]^+^, 189.0700 [M + H-C_6_H_10_O_2_]^+^ (Additional file 1), (Table 1). This metabolite was tentatively identified as primaquine quinone-imine carbamate based on accurate mass and fragments ions and may be generated through methylation, hydroxylation and quinone imine formation of carboxy PQ (Fig. 3).

### Plasma PQ metabolite profile

Very limited numbers of PQ metabolites were detected in plasma from the individuals treated with single dose of PQ. Besides PQ (*m/z* 260.1769) **(2)** and cPQ (*m/z* 275.1385) (**5**), only seven additional metabolites of PQ were detected in plasma. These include four phase I metabolites namely, hydroxy-primaquine quinone-imine (*m/z* 274.1549) (**1**), carboxyprimaquine lactam (*m/z* 257.1273) (**3**), Dihydroxy-carboxyprimaquine methylester (*m/z* 321.1454) (**8**) and carboxyprimaquine methyl ester (*m/z* 289.1560) (**6**) and three phase II metabolites, namely, glycosylated PQ (*m/z* 422.2315) **(7)**, PQ *N*-carbamoyl glucuronide (*m/z* 480.1992) (**4**) and PQ methyl carbamate (*m/z* 318.1789) (**9**). No hydroxylated metabolites of PQ were detected in plasma.

### Urine PQ metabolite profile

The urine from the individuals treated with PQ contained large numbers of PQ metabolites. PQ (*m/z* 260.1769) (**2**) and the major plasma metabolite cPQ (*m/z* 275.1385) (**5**) were detected in urine also. Besides these, the carboxy PQ lactam (*m/z* 257.1273) (**3**), predictably formed by spontaneous cyclization of carboxy side chain of cPQ and PQ carbamoyl glucuronide (*m/z* 480.1992) (**4**), a unique phase II metabolite formed through direct *N*-carbamoylation of PQ followed by glucuronidation, which were present in plasma, were also detected in urine. Glycosylated PQ (*m/z* 422.2315) (**7**), a prominent metabolite detected in plasma, was not detected in urine. Three acetylated metabolites were present in urine namely, PQ acetate (*m/z* 302.1875) (**29**), acetylated hydroxyl-PQ glucuronide conjugate (*m/z* 494.2127) (**20**) and *N*-Acetyl-PQ quinone-imine (*m/z* 316.1676) (**35**). Two metabolites with different retention times but identical accurate mass and MS/MS fragmentation profile corresponding to PQ glucuronide conjugate (*m/z* 436.2100) (**30, 34**) were also detected in urine.

Surprisingly, four mono hydroxylated metabolites of PQ, likely to be formed through CYP mediated pathways, were detected in urine. Three of these metabolites (*m/z* 276.1694) correspond to 2-OH (**18**), 3-OH (**21**), and 4-OHPQ (**14**), and the fourth (**17**) could be 7-OH, 8-*N*–OH, 1-*N*-oxide, or 8-*N*-oxide of PQ. Desmethy-PQ (*m/z* 246.1606) (**19**), and desmethyl-PQ glucuronide (*m/z* 422.1908) (**12**) were also detected in urine. Two metabolites predicted as PQ-quinone-imine (*m/z* 274.1549) (**1, 22**) and PQ-5,6-*ortho*-quinone (*m/z* 260.1403) (**10**), which is spontaneously generated from 5-OH-PQ, were also detected in urine. None of these phase I metabolites were detected in plasma. Further metabolism of hydroxyl-PQ was evident from presence of two dihydroxy-PQ (*m/z* 292.1647) metabolites (**25, 28**) and a dihydroxyl PQ quinone-imine (*m/z* 290.15) (**24**). Phase II conjugation metabolites, generated from further metabolism of hydroxyl PQ metabolites, detected in urine were two monohydroxy-PQ glucuronides (*m/z* 452.2011) (**11, 13**) trihydroxy-PQ glucuronide (*m/z* 484.1932) (**23**) and desmethyl-PQ glucuronide (*m/z* 422.1908) (**12**).

Further metabolism of cPQ, a major plasma metabolite, through phase I and phase II pathways of drug metabolism was evident from presence of several cPQ metabolites namely, cPQ glucuronide (*m/z* 451.1722) (**32**), hydroxyl cPQ glucuronide (*m/z* 467.1649) (**27**), trihydroxy-cPQ glucuronide (*m/z* 499.1545) (**26**) and dihydroxy cPQ methylester (*m/z* 321.1454) (**8**). Hydroxy-PQ alcohol (*m/z* 277.1559) (**33**), PQ alcohol glucuronide (*m/z* 437.1911) (**31**), hydroxyl-PQ alcohol glucuronide (*m/z* 453.1881) (**15**) detected in urine may be generated through further metabolism of PQ alcohol, the metabolite from oxidative deamination of PQ side chain alkyl amine.

## Discussion

Both therapeutic efficacy and toxicity of PQ have been attributed to its metabolites, likely generated through CYP mediated pathways of drug metabolism [10, 12]. Understanding of metabolism of PQ is highly critical for determining the mechanism of toxicity and efficacy of this drug [11, 12]. PQ has high value for global malarial control and eliminations efforts, due to its broad utility for treatment including radical cure, prophylaxis and prevention of malaria transmission [1–3, 33].

Based on the comprehensive LC-ESI-HRMS and MS/MS fragmentation profiles of PQ metabolites identified in plasma and urine of healthy human volunteers treated with single dose of 45 mg, PQ may be regarded as metabolized broadly through three pathways. Pathway 1 involves direct glucuronide/glucose/carbamate/acetate conjugation of PQ. The presence of *N*-carbamoyl glucuronide conjugate of PQ, both in plasma as well as in urine, indicates direct conjugation of PQ without its metabolism through phase I pathway. Generation of this metabolite involves *N*-carbamoylation of PQ by carbamoyltransferases, followed by glucuronide conjugation by UDP-glucuronosyltransferases (UGTs) [34]. Although this pathway is not widely appreciated in drug metabolism, a few drugs containing primary or secondary amines have been reported to undergo *N*-carbamoylation and glucuronidation [35, 36]. Incubation of such drugs with UGT under high CO_2_ tension yield *N*-carbamoyl glucuronide conjugates [36]. Mechanisms of this reaction involving human carbamoyltransferases and UGTs have not been identified yet. Metabolism of PQ through this pathway may be an important determinant for steady state concentration and overall pharmacokinetic-pharmacodynamics profiles of the drug. It was recently reported formation of *N*-carbamoyl glucuronide conjugate of PQ as a predominant metabolite, when PQ was incubated in vitro with primary human hepatocytes [20]. Formation of this metabolite in vitro with human hepatocytes was enantioselective, as (+)-*S*-PQ generated more than two-fold higher levels of the conjugate than (−)-*R*-PQ [20]. This may simply reflect the higher susceptibility of (−)-*R*-PQ to the oxidative deamination pathway [13]. Glycosylated PQ, another unexpected phase II PQ metabolite detected in plasma only, was also reported to form in vitro with primary human hepatocytes [20] and was more prominently formed from (+)-*S*-PQ than from (−)-*R*-PQ. In the human hepatocyte study, glycosylated PQ was detected at time zero and thought to be a simple adduct of the primary amine group of PQ and the aldehyde form of glucose via non-enzymatic means. This metabolite could also be formed in plasma by the same mechanism. Predominant metabolism of (+)-*S*-PQ through these pathways may explain the somewhat lower plasma concentrations of the (+)-*S*-PQ, despite its negligible metabolism through oxidative deamination to cPQ [13, 20].

The pathway 2 (Fig. 2) involves hydroxylation of PQ at different positions on the quinoline ring, forming mono-, di- and even tri-hydroxylated species, and subsequent conjugation of these ring hydroxylated metabolites to glucuronic acid. Surprisingly, none of the ring hydroxylated metabolites, 6-desmethyl PQ, or their glucuronide conjugates were detected in plasma. This may be due to rapid excretion of these metabolites in urine. It is interesting that the presumed active/toxic metabolite PQ-5,6-*ortho*quinone was only detected in urine but not in plasma. Recent PK and tissue distribution studies in mice showed rapid appearance of PQ-5,6-*ortho*quinone in high concentrations in the liver and in low concentration in plasma and its fast disappearance from the liver and circulation [37]. Formation of ring hydroxylated metabolites have also been demonstrated in vitro on incubation of PQ with primary human hepatocytes [20] and recombinant human CYP2D6 [21]. CYP2D6 and PQ incubations generated considerably high levels of ring hydroxylated PQ metabolites and 5,6-*ortho*quinone PQ. Earlier in vitro studies have indicated the role of multiple CYP isoforms in hemolytic toxicity of PQ with predominant involvement of CYP2D6 and CYP3A4 [10]. Subsequent reports have indicated the failure of PQ treatment in individuals with CYP2D6 poor metabolizer genotypes [50–52]. 5-Hydroxy metabolite, spontaneously converted to 5,6-*ortho*quinone metabolite, has been linked to therapeutic efficacy of PQ [53] as well as other 8-aminoquinolines namely tafenoquine and NPC1161B [54]. Recent in vitro studies with primary human hepatocytes and recombinant human CYP2D5 as well as in vivo experiments with mice have confirmed the formation of 5,6-*ortho*quinone through CYP2D6 mediated pathway [20, 21, 53].

The pathway 3 (Fig. 3) involves oxidative deamination of PQ at the terminal amine of the aminoalkyl side chain, resulting in formation of PQ-aldehyde, PQ alcohol and cPQ, which are further metabolized through additional phase I hydroxylations and/or phase II glucuronide conjugations. Rapid metabolism of PQ through monoamine oxidase-mediated oxidative deamination is responsible for short half-life of the drug. High circulating concentrations of cPQ, the major metabolite of this pathway, have been demonstrated in mouse [31], dog [38], primate [39] and human [13] pharmacokinetic studies. This metabolite is fairly rapidly cleared from the circulation in animals [31, 38, 39], while in humans high plasma levels of cPQ are maintained up to 24 h after PQ administration [13]. Even though cPQ is generally considered as an inactive and non-toxic metabolite, further metabolism of cPQ through phase I CYP-mediated pathways generate potential reactive ring hydroxylated metabolites and quinone-imine metabolites. Formation of ring-hydroxylated and quinone-imine metabolites of cPQ has also been demonstrated in vitro using primary human hepatocytes [18, 20]. The significance of these is not yet clear, but should be considered in interpretation of future studies.

This study presents the most comprehensive plasma and urinary metabolite profile of PQ in humans to date. The structures of metabolites were predicted on the basis of LC- profile, accurate mass and mass fragmentation patterns [18, 21, 22]. Identity of selected critical metabolites was also confirmed with the chemically synthesized standards [21, 22]. PQ is currently under large numbers of clinical trials in different populations in various countries, specially directed towards malaria control and elimination efforts [40–42]. Recent reports have indicated critical importance of pharmacogenetic factors, especially the role of CYP2D6 polymorphisms in therapeutic outcomes of PQ clinical trials. PQ is primarily used for *P. vivax* radical cure i.e. prevention of malaria relapse [1]. Recent global efforts have resulted in significant decline in deaths due to *P. falciparum* infections [43–45]. However, cases of *P. vivax* malaria have rather increased in some areas [46, 47]. PQ is also recommended for prevention of transmission of *P. falciparum* due to its activity against mature stage V gametocytes [48, 49]. PQ mass drug administration has also been suggested to hold promise in preventing malaria transmission for malaria control and elimination [40]. The risk of haemolytic toxicity of PQ in individuals with genetic deficiency of G6PD, which constitutes a significant proportion in malaria endemic countries, is a major concern [5]. Evaluation of PQ metabolite profiles in PQ clinical trials should be useful for better understanding of the variable therapeutic and safety profiles. The results reported in this paper provide useful baseline data for investigating PQ metabolite profiles in ongoing and future clinical studies with PQ and other 8-aminoquinolines. Direct metabolism of PQ through phase II conjugation pathway (pathway 1) and oxidative deamination (pathway 3), resulting in formation of cPQ as the most predominant metabolite, may determine pharmacokinetics and pharmacodynamics properties of PQ. Short half-life of PQ due to its metabolism to cPQ (presumed as an inactive metabolite) has been considered as a major limitation of PQ and requires 14 days’ long treatment for malaria radical cure [1]. Clinical trials with shorter 7 days’ treatment schedule have mostly yielded inclusive results. This study also reports further metabolism of cPQ through phase I reactions and requires attention regarding potential toxicity of this metabolite. No metabolite has still been unequivocally linked to efficacy and/or toxicity of PQ. However, besides PQ, determination of key metabolites of PQ from each pathway namely PQ *N*-glucuronide, 5,6-*ortho*quinone PQ and cPQ along with genotyping for CYP2D6 in PQ clinical trials may help for finding the link between PQ metabolism and therapeutic outcomes.

## Conclusion

This is the most exhaustive study on identification and characterization of human metabolites of primaquine, the only approved drug for malaria radical cure. Based on overall metabolites’ profile, three pathways were predicted for metabolism by humans, (a) direct glucuronide/glucose/carbamate/acetate conjugation of PQ (b) hydroxylation of PQ at different positions on the quinoline ring and (c) oxidative deamination of PQ at the terminal amine of the aminoalkyl side chain. Metabolism of PQ through direct conjugation and oxidative deamination pathways may determine pharmacokinetics and pharmacodynamics of this important antimalarial drug. Metabolism of PQ through hydroxylations on the quinolone ring, predictably catalyzed by cytochrome P_450_ (CYP2D6) mediated pathways, generates monohydroxylated PQ metabolites. These metabolites have been implicated in therapeutic efficacy and hemolytic toxicity of PQ. Further, extension of these studies to quantitative profiling of key metabolites in malaria infected individuals treated with PQ and their correlation with therapeutic outcomes *vis*-*à*-*vis* CYP2D6 genotypes may be useful in determining the rational approach for application of PQ in malaria radical cure, the primary application of this drug, as well as malaria control programs.

## Additional file

**Additional file 1: Fig. 1S.** Key fragments of PQ and other metabolites using LC/ESI-QToF

## Abbreviations

PQ: primaquine
cPQ: carboxyprimaquine
UHPLC-QToF-MS: ultra high-performance liquid chromatography coupled with high-resolution mass spectrometric
G6PD: glucose 6-phosphate dehydrogenase
HRMS: high resolution mass spectrometry
