# Supplementary material for: Metabolism of primaquine in normal human volunteers: investigation of phase I and phase II metabolites from plasma and urine using ultra-high performance liquid chromatography-quadrupole time-of-flight mass spectrometry
Source: Malar J. 2018 Aug 13;17:294. doi: 10.1186/s12936-018-2433-z (PMC6090659; doi:10.1186/s12936-018-2433-z)
Supplement: Supplementary file 1 — Additional file 1: Fig. 1S. Key fragments of PQ and other metabolites using LC/ESI-QToF [file 12936_2018_2433_MOESM1_ESM.docx]

**Supporting Data**

**Metabolism of Primaquine in Normal Human Volunteers: Investigation of Phase I and Phase II Metabolites from Plasma and Urine using Ultra-High Performance Liquid Chromatography-Quadrupole Time of Flight Mass Spectrometry**

Bharathi Avula^1^, Babu L. Tekwani^1,2*^, Narayan D. Chaurasiya^1^, Pius Fasinu^1^, NP Dhammika Nanayakkara^1^, HMT Bhandara Herath^1^, Yan-Hong Wang^1^, Ji-Yeong Bae^1^, Shabana I. Khan^1,2^, Mahmoud A. Elsohly^1,3^, James D. McChesney^4^, Peter A Zimmerman^5^, Ikhlas A. Khan^1,2^ and Larry A. Walker^1,2^

^1^National Center for Natural Products Research, Research Institute of Pharmaceutical Sciences, School of Pharmacy, The University of Mississippi, University, MS 38677, USA;  ^2^Division of Pharmacology, Department of BioMolecular Sciences, School of Pharmacy, The University of Mississippi, University, MS 38677, USA; ^3^ElSohly Laboratories, Incorporated, 5 Industrial Park Drive, Oxford, MS 38655, USA; ^4^Ironstone Separations, Inc, 147 CR 245, Etta, MS 38627, USA; ^5^Center for Global Health & Diseases, Case Western Reserve University Cleveland, Ohio 44106 USA.

*Corresponding author

Email Address: btekwani@olemiss.edu

Telephone #: 1-662-915-7882; Fax #: 1-662-915-1006

**Fig. S1 Key fragments of PQ and other metabolites using LC/ESI-QToF**

**(1, 22) Primaquine quinone-imine (Peak 274)**

**(2) Peak 260 (precursor compound PQ) (LC-MS/MS profile identical to synthetic standard)**

**(3) Peak 257 (Carboxyprimaquine lactam) (LC-MS/MS profile identical to synthetic standard)**

**(4) Peak 480 (Primaquine *N*-carbamoyl-glucuronide) (LC-MS/MS profile identical to synthetic standard)**

**(5) Peak 275 (Carboxyprimaquine) (LC-MS/MS profile identical to synthetic standard)**

**(6) Peak 289 (Carboxyprimaquine methyl ester) (LC-MS/MS profile identical to synthetic standard)**

**(7) Peak 422 (Primaquine glucose conjugate) (LC-MS/MS profile identical to synthetic standard)**

**(9) Peak 318 (Primaquine methyl carbamate) (LC-MS/MS profile identical to synthetic standard)**

**(10) Peak 260 (Primaquine-5,6-*ortho*-quinone)**

**(12) Peak 422.1908 [M+H]^+^  (Desmethyl primaquine-*O*-glucuronide)-** Tentatively identified based on accurate mass and fragments ions

**(14, 17, 18, 21) Peak 276 (Monohydroxy primaquine)- 14, 18 & 21 were accurately identified as 4-OH, 2-OH and 3-OH with LC-MS/MS profile identical to synthetic standards.**

**(19) Peak 246 (Desmethyl primaquine)-** Tentatively identified as based on accurate mass and fragments ions

**(20) Peak 494 (Acetylated hydroxyl primaquine glucuronide)-** Tentatively identified as based on accurate mass and fragments ions

**(24) Peak 290 (Monohydroxy primaquine quinone-imine)-** Tentatively identified as based on accurate mass and fragments ions

**(25, 28) Peak 292.1647 [M+H]^+^  (Dihydroxy primaquine)-** Tentatively identified as based on accurate mass and fragments ions

**(26) Peak 499 (Trihydroxy carboxyprimaquine glucuronide)-** Tentatively identified as based on accurate mass and fragments ions

**(27) Peak 467 (Hydroxy carboxyprimaquine glucuronide)-** Tentatively identified as based on accurate mass and fragments ions

**(29) Peak 302 (Primaquine *N*-acetate)- LC-MS/MS profile identical to synthetic standard**

**(31) Peak 437 (Primaquine alcohol glucuronide)-** Tentatively identified as based on accurate mass and fragments ions

**(32) Peak 451 (Carboxy primaquine glucuronide)- LC-MS/MS profile identical to synthetic standard**

**(33) Peak 277 (Hydroxy primaquine alcohol)-** Tentatively identified as based on accurate mass and fragments ions

**(35) Peak 316 (*N*-Acetyl primaquine quinoneimine)-** Tentatively identified as based on accurate mass and fragments ions

**(36) Peak 303 (Carboxyprimaquine iminoquinone methyl ester)-** Tentatively identified as based on accurate mass and fragments ions
